# A Comprehensive Review of Infectious Granulomatous Diseases of the Gastrointestinal Tract

**DOI:** 10.1155/2021/8167149

**Published:** 2021-02-06

**Authors:** Shivantha Amarnath, Liliane Deeb, Jobin Philipose, Xiaomin Zheng, Vivek Gumaste

**Affiliations:** ^1^Department of Internal Medicine, Staten Island University Hospital, Northwell Health, Staten Island, NY, USA; ^2^Department of Gastroenterology and Hepatology, Staten Island University Hospital, Northwell Health, Staten Island, NY, USA; ^3^Department of Pathology, Staten Island University Hospital, Northwell Health, Staten Island, NY, USA

## Abstract

A granuloma is defined as a localized inflammatory reaction or a hypersensitive response to a nondegradable product leading to an organized collection of epithelioid histiocytes. Etiologies of granulomatous disorders can be divided into two broad categories: infectious and noninfectious (autoimmune conditions, toxins, etc.) causes. The endless list of causalities may prove challenging for gastroenterologists and pathologists to formulate a list of clearly defined differentials. This is true when distinguishing these etiologies based on various clinical presentations and endoscopic and histological findings. We aim to provide a comprehensive review of some of the frequent and rare infectious granulomatous diseases of the gastrointestinal tract documented in the literature to date. We provide an overview of each infectious pathology with an emphasis on epidemiology, clinical presentation, and endoscopic and histologic findings, in addition to treatment.

## 1. Introduction

A granuloma can be described as a localized inflammatory reaction or a hypersensitive response to a persistent foreign entity leading to an organized collection of epithelioid histiocytes, a key feature of granulomas. This histological appearance ranges from small clusters of histiocytes, as seen in Crohn's disease, to huge well-circumscribed whorls of cells commonly appreciated in Sarcoidosis [1]. A layer of histiocytes around a pool of necrotic debris is seen in tuberculosis and fungi. The presence of giant cells is helpful but not essential for diagnosis.

Granulomas can have either necrotizing or nonnecrotizing features. Caseating granulomas, also referred to as necrotizing granulomas, are aptly named for their gross appearance of necrosis similar to cheese. Etiologies of granulomatous disorders can include bacterial, fungal, or parasitic infections; autoimmune diseases; and certain toxins or irritants. The endless list of causalities may prove challenging for gastroenterologists and pathologists to formulate a list of clearly defined differentials. This is true when distinguishing these etiologies based on various clinical presentations and endoscopic and histological findings.

A detailed step-wise approach to determine a granuloma's etiology from a gastrointestinal (GI) biopsy is discussed separately in the article by Brown and Kumarasinghe [2]. Briefly, when a granuloma is discovered on biopsy, the initial step is to classify if the granuloma has caseating or noncaseating properties. Caseating granulomas are commonly encountered in infectious etiologies and are typical for tuberculosis. If an infection is suspected, special stains such as Grocott's Methenamine Silver (GMS) stain and acid-fast stain can aid in the diagnosis. However, cultures remain the gold standard. Many noninfectious etiologies contribute to granulomas, including autoimmune disorders (Crohn's disease, Sarcoidosis, foreign body reactions, and occasionally lymphomas). The review primarily focuses on infectious granulomas that are encountered within the alimentary tract. Tables 1, 2, and 3 summarize the common infective sites and key diagnostic (endoscopic and histologic) findings of the various infectious causes of granulomas seen in the GI tract.

## 2. Methods

A comprehensive literature search was performed using PubMed, MEDLINE, Embase, and Cochrane databases. The protocol for evidence research was established using a combination of validated “Medical Subject Headings” (MeSH^Ⓡ^) terms: granuloma, gastrointestinal tract (upper and lower), infections, bacterial infections, fungi, and parasites. This was followed by an in-depth literature review of the various pathogens uncovered using these search terms. To avoid the exclusion of key differentials, we performed a second literature search for infectious granulomas (using the above stated search terms) based on the broad list of differentials in the review articles by Brown and Kumarasinghe and James [2, 3]. Our final list of pathogens responsible for granuloma formation in the GI tract based on our literature search included Mycobacterium tuberculosis, Yersinia infections, lymphogranuloma venereum, Histoplasmosis, Coccidioidomycosis, Cryptococcosis, Schistosomiasis, Anisakiasis, Bartonella henselae or cat scratch disease, Salmonellosis, syphilis, Basidiobolomycosis, and Leishmaniasis. All full-text articles (ranging from case reports, case series, literature reviews, to observational studies) published in the English language over the last 20 years (2000-2020) involving human subjects were included in our review. This article is aimed at providing an up-to-date comprehensive overview of the various infectious etiologies of granulomas seen in the gastrointestinal tract that are frequently documented in the literature.

### 2.1. Bacterial Causes of Granulomas in the Gastrointestinal Tract

#### 2.1.1. Mycobacterium tuberculosis

Tuberculosis (TB) is a disease caused by *Mycobacterium tuberculosis*, an aerobic, acid-fast bacillus with a preference for lung tissue [4, 5]. An estimated 1/3 of the World's population is affected by TB, and approximately 9 million new cases are reported each year [5–8]. Intestinal TB [4] represents the sixth most common extrapulmonary location and may manifest as a primary gastrointestinal infection or secondary to reactivation or spread from a pulmonary focus [5–8]. Extrapulmonary TB is most prevalent among females, non-Hispanic blacks, and HIV-positive individuals. However, statistical data specific to intestinal tuberculosis (ITB) are limited [9, 10].

ITB presents with nonspecific symptoms such as fever, night sweats, weight loss, abdominal pain, nausea, vomiting, weakness, rectal bleeding, and diarrhea [11, 12]. A recent meta-analysis, published in 2017, involving 3,706 patients, focused on specific parameters for identifying ITB compared to a common imitator, Crohn's disease (CD). This study found that fever, night sweats, pulmonary symptoms, and ascites were the most significant findings distinguishing ITB from CD [13]. The gastrointestinal manifestations of ITB are contingent upon the location of the involvement. Therefore, esophageal TB presents with odynophagia, hematemesis, and dysphagia and is commonly mistaken for malignancy. Gastric and duodenal TB may present like peptic ulcer disease [5, 12]. Ileocecal and rectal TB are frequently confused for CD. Colonic TB can mimic ulcerative colitis (UC) or malignancy [5]. ITB usually affects one single location, and the most commonly affected area is the ileocecal region. One possible explanation is due to the slow transit and relatively static physiological environment that occurs in the ileum, which allows for prolonged contact between the bacillus and mucosa [5, 12, 14, 15]. Complications can include obstruction with perforation, fistulization, bleeding, and malabsorption [12]. In the case of fistulas, perforation, hemorrhage, obstruction, abscess, or sphincter involvement, surgery may be needed [5, 11, 12].  Table 4 summarizes the histological features that distinguish TB from Crohn's disease and *Yersinia* spp.

A variety of imaging modalities are available to diagnose ITB. ITB and concomitant pulmonary TB occur 15-20% of the time. Therefore, chest radiographs are positive and helpful for diagnosis in a minority of patients [12, 15]. Abdominal radiographs are nonspecific but are often used to assess for potential complications such as obstruction or perforation [15]. Ultrasound can detect ascites, and the aspirate can be measured for adenosine deaminase levels, which is very sensitive and specific. However, staining and culture have lower sensitivity [5, 6, 12, 16]. CT and MRI modalities can show concentric wall thickening with a narrowed lumen, an agape ileocecal valve, and strictures with prestenotic dilation. Enlarged lymph nodes with peripheral enhancement and radiolucent centers representing inflammatory changes surrounding central necrosis are common in ITB and rarely observed in CD [5, 12, 17].

Ileocolonoscopy with biopsy remains invaluable to the diagnosis of ITB [12, 15]. Gross findings have been classified into three types: ulcerative, hypertrophic, and ulcerohypertrophic. Pseudopolyps, nodules, circumferential transverse ulcers, and a deformed ileocecal valve are frequently seen [6, 15, 17]. These findings may also be seen in CD but are less common compared to ITB [18]. Upper GI involvement with a negative ileocolonoscopy may warrant gastroduodenoscopy or enteroscopy to assess for transverse ulcers or hypertrophic nodular lesions [6, 17].

**Figure 1:**
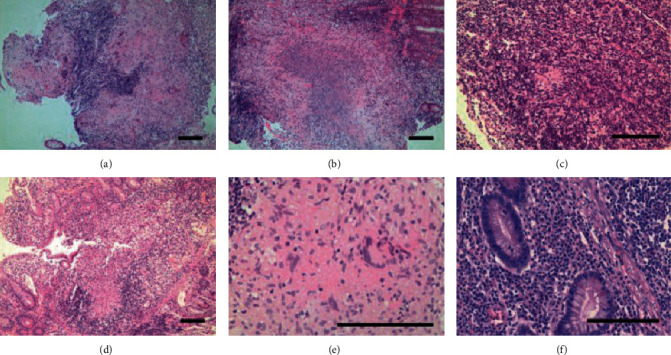
Histological features of intestinal tuberculosis (ITB) and Crohn's disease (CD) discovered in endoscopic biopsy specimens. ITB commonly features several large, confluent caseating granulomas (a), with central necrosis (b) and giant cells (e). CD features relatively few, small granulomas (c). Large granulomas in CD are rare, and if present, they are poorly organized (d). Basal plasmacytosis can be seen in CD (f). (Images obtained with permission from Ye et al. [20].

Biopsies obtained from masses and ulcer margins should be stained for acid-fast bacilli (AFB), scanned for caseating granulomas, and sent for culture. These techniques are considered the most accurate for ITB; however, positive AFB occurs only 25-36% of the time, granulomas are seen in 50-80% of biopsies, and cultures take 4-6 weeks to return. Clinicians may find themselves attempting to diagnose ITB without these results [6, 12]. When ITB granulomas are seen, they are multiple, large, confluent, and caseating, as seen in Figure 1 [19, 20]. Five or more granulomas per ten low power fields or a diameter greater than 400 micrometers favors ITB [20]. Other studies suggest that granulomas greater than 500 micrometers in size and the presence of ten or more granulomas accurately indicated a diagnosis of ITB [19, 21]. Other features such as epithelioid histiocyte lined ulcers, submucosal granulomas, lymphocyte cuffing, and excessive submucosal inflammation favored ITB over CD [13, 21].  Table 4 summarizes the key histological features that help distinguish ITB from CD. Features that were found to be nonspecific were the presence of acute or chronic inflammation and distortion of tissue architecture [21]. Recently, CD73 was found to be a marker for mesenchymal cells in ITB granulomas but not in CD granulomas [22]. However, cultures are still the best confirmatory test for TB, although it takes time. The tissue histology for TB is not sensitive, but evidence of necrotizing granuloma and positive AFB stain is highly specific (97.5%) for diagnosis.

Interferon-gamma release assay (IGRA) can be useful in the diagnosis of ITB. A meta-analysis found that IGRA has a mean sensitivity and specificity of 74% and 87%, respectively, for ITB [22]. Molecular techniques such as PCR have a 93% specificity in detecting ITB from biopsy samples [20]. PCR's overall sensitivity for mycobacterium is not high (47%), and PCR is generally performed only on Auramine-Rhodamine-positive specimens [23].

In the absence of confirmatory testing or if the diagnosis is still equivocal, then as a last resort, a therapeutic trial of anti-TB medication may be warranted, and clinical response can be monitored [6]. This approach is considered safe, as the misdiagnosis of ITB as CD and treatment with steroids can be fatal [19]. Treatment principles of ITB have been inferred from the treatment of pulmonary TB with the use of isoniazid, rifampin, ethambutol, and pyrazinamide. Studies did not show any difference in the treatment duration between six or ninth months and recommend a total treatment duration of six months [24]. If symptoms fail to resolve with antibiotics, it may indicate a misdiagnosis of CD or drug-resistant TB [17]. Therefore, endoscopic reassessment for any mucosal healing may aid in the diagnosis [19].

#### 2.1.2. Yersinia Bacterium

Yersinia is a pleomorphic Gram-negative coccobacillus that commonly affects the pediatric population below 10 years of age [25]. *Y. enterocolitica* is the most common species causing yersiniosis [26]. They can cause mesenteric lymphadenitis, appendicitis (a common cause in the western hemisphere), and ileocolitis (mimicking inflammatory bowel disease) [26]. The incidence of this infection increases in the winter season [27]. Other gastrointestinal complications include perforation, intussusception, subacute obstruction, and hepatic abscesses [25] [27]. Systemic manifestations may include septic arthritis, encephalitis, Reiter's syndrome, erythema nodosum, and reactive polyarthritis [26].

Transmission is mostly via the consumption of contaminated water or food, especially pork products since pigs are the favored reservoir [25, 26]. The incubation period is usually 24-48 hours after ingestion. Intestinal yersiniosis mostly presents with diarrhea (87%), fever (76%), abdominal pain (47%), and vomiting (31%) [26]. The most common locations affected include the ileum, cecum, appendix, and mesenteric lymph nodes [25, 27, 28].

The best diagnostic modality for yersiniosis is PCR [26], but antibody titers must be obtained [20]. CT of the abdomen will show signs of colitis, ulceration, and sometimes aphthoid ulcers in the terminal ileum with or without mesenteric lymphadenopathy and appendiceal inflammation [25, 27]. These ulcers have a high susceptibility to bleeding and can lead to iron deficiency anemia [25]. It can also present as a pseudotumor on imaging, and this is frequent in *Y. pseudotuberculosis* [25]. Endoscopic findings can include signs of acute inflammation in the distal ileum and cecum together with purulent or necrotic lymphadenopathy [27].

**Figure 2:**
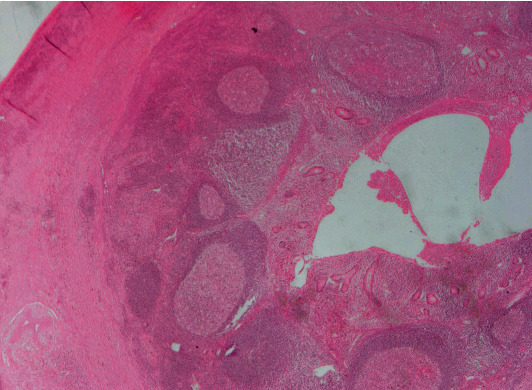
Histopathological specimen of a case of appendicitis caused by *Yersinia* spp. demonstrates lymphoid hyperplasia and suppurative granulomas. (Image obtained with permission from Weisenberg [30].)

Biopsy findings of the appendix and terminal ileum will demonstrate suppurative epithelioid granulomas with prominent lymphoid cuffs, typical of *Y. enterocolitica* infection, as shown in Figure 2 [28]. Other findings include transmural inflammation and cryptitis hence its similar histology to CD [29]. However, the presence of suppurative granuloma and a positive serology will favor the diagnosis of Yersinia (Figure 2) [29]. Yersiniosis is self-limiting, and if antibiotic use is required, it responds best to fluoroquinolones alone or in combination with cephalosporins [25].

#### 2.1.3. Lymphogranuloma Venereum

Lymphogranuloma venereum (LGV) is a known cause of proctitis and occurs almost exclusively in men who have sex with men (MSM), especially amongst those infected with HIV or HCV [31–33]. HIV is evident in >70% of LGV cases, even in the absence of overt immunosuppression [33]. There is an ongoing theory that in the region of the rectal mucosa, LGV is mostly dependent on the T cell-mediated immune response, thus explaining its higher predominance in HIV-infected individuals. LGV is endemic to Africa, the Caribbean Islands, and Southeast Asia [33]. *Chlamydia trachomatis* serovars L1-3 are responsible for the inflammatory changes seen in proctitis, and L2B is the most common in the western hemisphere [34].

Clinical presentation and biopsy findings of LGV are almost indistinguishable from IBD and are often misdiagnosed. However, painful bilateral inguinal lymphadenopathy is unique to LGV. It should be suspected in any HIV patient showing symptoms related to IBD, patients with proctitis, or those not responding to IBD therapy [33]. LGV's presentation has three main stages [33]: The primary stage is apparent 3-30 days after transmission and often presents as nontender papules in the genital region that often ulcerate; The second stage occurs three weeks after proctitis and presents as anal pruritis with discharge, pain, and tenesmus. “Buboes” is a term often given to any evidence of unilateral or bilateral lymphadenopathy. The final stage involves chronic inflammation and the formation of fibrotic tissue, strictures, and fistulas, especially in the colorectal region. It is imperative to acquire a good sexual history in any HIV patient complaining of GI symptoms. A physical exam will show evidence of stenotic and tender anal canal [33].

**Figure 3:**
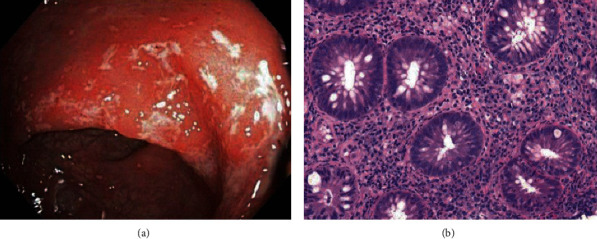
Endoscopic view of an HIV patient diagnosed with lymphogranuloma venereum (LGV) proctitis demonstrates moderate colitis in the rectum with erythematous and friable mucosa (a). Rectal biopsy specimen highlights moderately active proctitis with focal crypt distortion and cryptitis similar to CD (b). (Images obtained with permission from Gallegos et al. [35].

LGV is primarily diagnosed using RT-PCR of a rectal swab, which helps identify the serovar subtype. Alternatively, testing for elevated anti-Chlamydia antibody titers can also be diagnostic [33]. Once detected, a full sexually transmitted disease (STD) panel and HIV testing should be sent [31]. Endoscopic findings are often similar to IBD and will demonstrate chronic inflammatory changes with strictures and ulcerative lesions in the mucosa and are usually covered with pus, blood, and granulation tissue [33]. Biopsy findings may vary depending on the stage of inflammation. Acute inflammation may show signs of cryptitis and crypt abscesses, but crypt distortion is often minimal, unlike ulcerative proctocolitis, where architectural changes are prominent (Figure 3). Chronic inflammation will demonstrate granulomatous inflammation with focal necrosis and a tenfold increase in crypt abscesses [32, 33].

Rectal LGV is managed using doxycycline 100 mg twice daily for three weeks. Alternate options include oral tetracycline 500 mg four times daily for three weeks or azithromycin 1 g per week for three weeks [33]. Surgery is often reserved for any complications of LGV. It is vital to treat the sexual partner if the sexual contact was within 60 days before the onset of symptoms, with either a single dose of azithromycin or doxycycline twice daily for seven days.

### 2.2. Fungal Infectious Granulomas of the Gastrointestinal Tract

#### 2.2.1. Histoplasmosis

Histoplasmosis is a saprophytic dimorphic fungus that grows as a mold in the environment and yeast in physiological tissue and culture media [36]. It is present worldwide and is endemic to the Mississippi and Ohio River valleys in the USA. It is 2-4 *μ*m in size and is transmitted as spores predominantly affecting the lung parenchyma via direct inhalation. In healthy individuals, it is primarily limited to the respiratory tract. However, almost all GI Histoplasmosis are due to disseminated disease, and on rare occasions, it can present as a direct gastrointestinal infection via the consumption of contaminated water [37]. Its notoriety is common amongst immunocompromised hosts, including patients with AIDS (CD4 < 150 cells/mm^3^), lymphomas, chronic diseases, and the elderly [38]. In these patients, it presents as a disseminated systemic pathology due to hematogenous spread.

**Figure 4:**
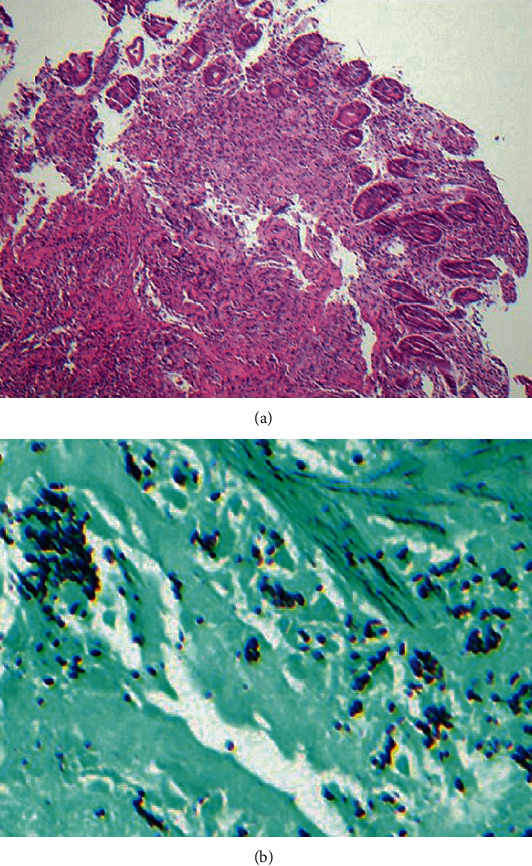
Hematoxylin and eosin (H&E) stain of a small intestinal biopsy specimen demonstrates granulomatous inflammation in an HIV patient diagnosed with disseminated Histoplasmosis (a). GMS stain reveals macrophages with intracellular fungal organisms (b). (Images obtained with permission from Spinner et al. [43]).

GI involvement is present in 70-90% of disseminated cases but is not always the initial manifestation [36]. Common symptoms include fever, weight loss, abdominal pain, distention, and, most importantly, hepatosplenomegaly. The most frequently infected site is the ileocecal junction secondary to the abundance of lymphoid aggregates in this region. This is then followed by the colon and, occasionally, the small intestine and upper GI tract [36]. Regional lymphadenopathy is a frequent theme in these affected sites and is often present in almost all cases of Histoplasmosis [36, 39]. Recently, four distinct pathologies have been described [36]: subclinical and microscopic clusters of macrophages localized in the lamina propria; plaques and pseudo polyps resulting from fungi containing macrophages; tissue necrosis and ulcerative lesions causing clinical manifestations such as abdominal pain, diarrhea, and bleeding; and localized thickening and acute inflammation of the bowel, consequently mimicking CD. Abdominal CT imaging commonly demonstrates polypoid lesions in affected sites and, in some cases, apple core lesions in ascending and transverse colon with surrounding lymphadenopathy [36]. Colonic skip lesions are not exclusive to CD and are also frequent in infectious colitis [40]. Colonoscopic findings include friable polypoid masses with focal erosions ranging from the terminal ileum to the rectum [41]. Biopsy commonly highlights caseating granulomas containing intracellular yeast with narrow buds that stains positive for PAS and GMS silver stains (Figure 4). However, Histoplasmosis can also present as noncaseating granulomas but appear fewer in number (<2 granulomas per slide) with ragged borders. This is in contrast to Sarcoidosis, which demonstrates numerous granulomas (>10 per slide) with clearly defined, sharp borders [34, 42].

Complications of GI Histoplasmosis include obstruction, perforation, and hemorrhage and may require surgical intervention. When massive lower GI bleeding is suspected secondary to infectious colitis, Histoplasmosis should always be considered, and it will often be coinfected with Entamoeba and Cytomegalovirus (CMV). It has been proposed that the amoebic parasite can contribute to the virulence and pathogenic trait of Histoplasmosis [44].

GI Histoplasmosis is clinically managed via induction therapy with amphotericin B for two weeks, followed by long-term management with itraconazole [36]. If it is coinfected with Entamoeba, then metronidazole should be used as an adjunctive therapy. Similarly, ganciclovir is used for CMV coinfection. Untreated Histoplasmosis infection is fatal, with mortality rates of 83%, and with treatment, this can be reduced to 26% [45]. Additionally, it should be noted that Histoplasmosis can be latent in the terminal ileum in immunocompetent individuals, only to resurface as a result of immunosuppression.

#### 2.2.2. Coccidioidomycosis

Coccidioidomycosis, also referred to as Valley fever, is endemic to Southwestern USA and is transmitted via inhalation of spores (*Coccidioides immitis* or *Coccidioides posadasii*) [46]. It is often limited to the pulmonary system. Systemic manifestation is infrequent, and the GI system is presumed to be spared [46–48]. Risk factors for dissemination include immunocompromised state, third trimester of pregnancy, male predominance (2 : 1), and African-American and Filipino ethnicities [46, 47]. There is also a reported case of association between Coccidioidomycosis and blood group B [47].

**Figure 5:**
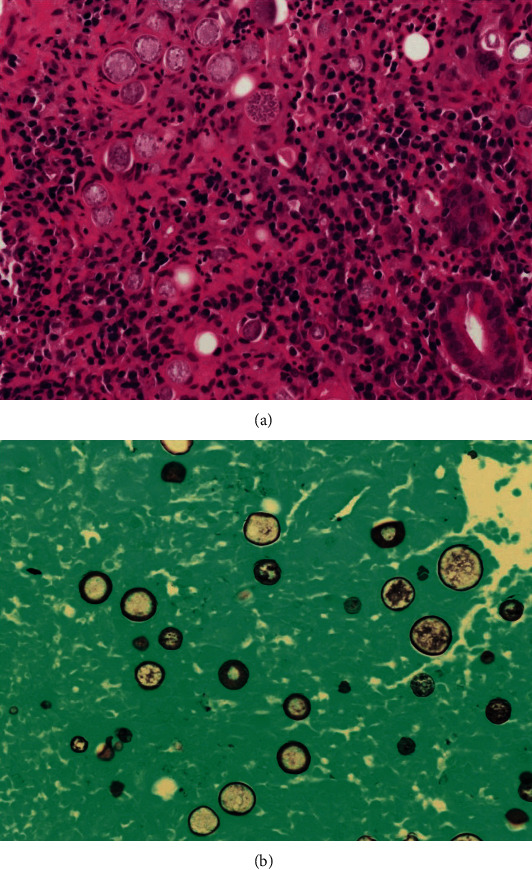
Duodenal biopsy shows multiple spherules filled with round fungal endospores, and dispersed isolated endospores can be seen in the lamina propria on H&E stain (a) and GMS stain (b). (Images obtained with permission from Zhou et al. [46]).

As mentioned previously, GI manifestations are infrequent at <1% [49]. However, there are more than 30 reported cases of peritoneal Coccidioidomycosis, which stated to occur due to hematogenous spread from a lung foci or ingestion of pulmonary secretions [47]. This is often misdiagnosed as a primary lung malignancy with peritoneal carcinomatosis [49]. Hence, it is imperative to acquire a good history from the patient. Peritoneal Coccidioidomycosis often presents with abdominal distention followed by abdominal pain, hernia, and fever [47]. CT of the abdomen shows evidence of omental thickening with areas of loculated ascites. It is possible to find multiple white plaques distributed throughout the peritoneum and omentum on diagnostic laparoscopy [47]. Some cases report the localization of the pathogen in the duodenum and are proposed to be associated with complications of perforation and obstruction [46, 48]. Biopsies demonstrate caseating, suppurative, and granulomatous inflammation with giant cells that attempt to destroy multinucleated thick-walled spherules filled with endospores. It should be noted that these endospores can rupture, causing further dissemination (Figure 5). Histologically, Histoplasmosis and Cryptococcosis can be distinguished from *Coccidioides* spp. based on variation in the endospore size, growing spherule, and the lack of narrow-based budding [50].

A diagnosis can be made in one of 3 ways [46]: identification of spherules in cytology or biopsy, the culture of any bodily fluids positive for Coccidioidomycosis, or positive serology for antibodies against the pathogen's antigens.

Patients with nondisseminated Coccidioidomycosis are managed with azole antifungal therapy [47]. However, further research is required to determine optimal therapy for peritoneal Coccidioidomycosis.

#### 2.2.3. Cryptococcosis

Cryptococcus is a yeast-like fungus that is frequently found in soil and bird droppings [51]. It most commonly presents as an opportunistic infection affecting HIV patients with CD4 counts < 100 cells/mm^3^ and other immunocompromised individuals [51]. Initial clinical manifestation includes meningitis, and the respiratory and GI system are infrequently involved [52–54]. The mode of infection of the GI tract can either be due to hematogenous dissemination or direct inoculation during surgical procedures involving the abdomen [54]. However, Girardin et al. reported a rare case of cryptococcal gastroduodenitis as the initial presentation in an HIV-infected patient [52]. Chronic abdominal pain in an AIDS patient is most commonly secondary to TB, CMV, or Cryptosporidium. However, several studies have reported that if HIV patients develop abdominal pain immediately after the commencement of antiretroviral therapy (ART), opportunistic infections such as Cryptococcus should be considered in the differential. This is primarily due to IRIS that can arise as a complication from ART [53].

**Figure 6:**
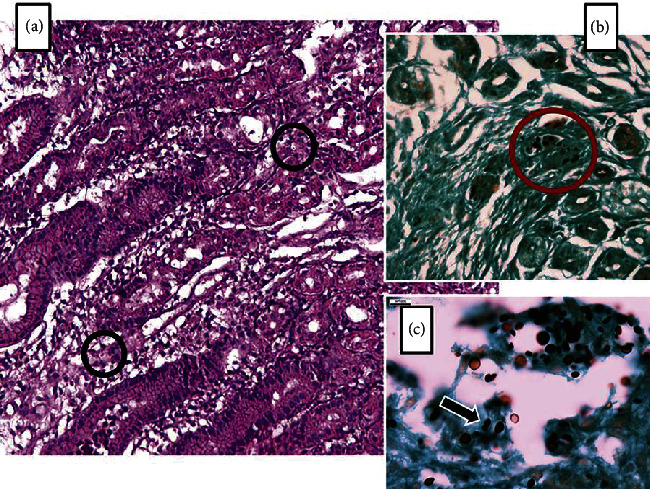
Histopathological specimen of an HIV patient diagnosed with gastric Cryptococcosis. An H&E stain demonstrates oxyntic glands in the body of the stomach with significant edema and lymphoplasmacytic inflammation of the lamina propria (a). Different-sized round structures with a halo clearing of each pathogen can be seen (black circles). GMS staining demonstrates various sized yeast-like structures (red circle in (b)). Higher-resolution GMS staining (×400) highlights organisms with a narrow-based budding consistent with *Cryptococcus* spp. (c). (Images obtained with permission from Eyer-Silva et al. [55]).

Cryptococcus usually affects the upper GI system with preference to the duodenum and esophagus [51–54]. Hence, upper endoscopy is the best tool for diagnosis [52]. Biopsy findings vary depending on whether or not patients are on ART [53]: biopsies of patients not on ART will mostly show yeast proliferation with a moderate histiocytic response and minimal recruitment of lymphocytic and neutrophilic components (Figure 6) [53]. In those receiving ART, biopsies will show CD4 T cells and a larger number of histiocytes and multinucleated giant cells [53]. Additionally, there may be signs of atrophic intestinal mucosa with neutrophilic and eosinophilic polynuclear infiltrate within the lamina propria. The pathogen will stain positive for PAS, GMS, and Alcian blue [52, 54]. Staining with Alcian blue signifies the production of mucopolysaccharides by the Cryptococcus pathogen [52].

Disseminated Cryptococcus is managed with amphotericin B and flucytosine induction therapy for two weeks, followed by chronic management with fluconazole. The optimal treatment for the GI Cryptococcus requires further research.

### 2.3. Parasitic Infectious Granulomas of the Gastrointestinal Tract

#### 2.3.1. Schistosomiasis

Schistosomiasis is primarily caused by the genera *S. japonicum*, *S. mansoni*, and *S. haematobium*, *S. mansoni*, *S. japonicum*, *S. intercalatum*, and *S. mekongi* which are mostly responsible for intestinal and hepatosplenic Schistosomiasis [56]. It is the second most frequent tropical disease after malaria [56]. The African subcontinent, South America, and Southeast Asia are the most Schistosomiasis-prevalent locations. The incidence of Schistosomiasis has steadily increased to neighboring countries due to immigration [56]. The life cycle of Schistosomiasis is complex. Briefly, Schistosoma eggs hatch and infect snails, which are the intermediate hosts. The snails release Cercariae into the water, which penetrates a mammalian host's skin and becomes Schistosomula. These initially reach the lung and then enter the hepatoportal circulation and become mature adults and begin mating. They subsequently migrate to other tissues depending on the type of Schistosome and inhabit the surrounding veins. For instance, *S. japonicum* infects the mesenteric veins and drains into the small intestine. *S. mansoni* prefers the superior mesenteric veins and empties into the large intestine. The females lay eggs that are antigenic and trigger an inflammatory response in the host, resulting in a granuloma formation around the egg. This facilitates the transmigration of eggs to enter the lumen. Excretion of eggs in the feces completes the parasitic life cycle [57].

Patients may initially present with a light rash at the site of Cercariae penetration. They also present with Katayama fever characterized by fever, fatigue, and dry, nonproductive cough for 4-12 weeks as a systemic manifestation migrating morulae [57]. GI symptoms are broad but mostly involve nonspecific generalized abdominal pain, transient dysentery. <10% of patients develop hepatosplenic disease characterized by hepatosplenomegaly, ascites, and portal hypertension.

Diagnosis of intestinal Schistosomiasis can be made from routine blood work as well as proper history taking. Laboratory studies may reveal eosinophilia, iron deficiency anemia, thrombocytopenia, and transaminitis. Direct endoscopic visualization of the small and large intestines can include the following [58]: early findings can include mucosal edema with petechial hemorrhage and ulceration of the bowel wall, especially in the right colon. The biopsy will show congestive mucosa with Schistosoma ova within the lamina propria with submucosal infiltration of eosinophils and neutrophils.

**Figure 7:**
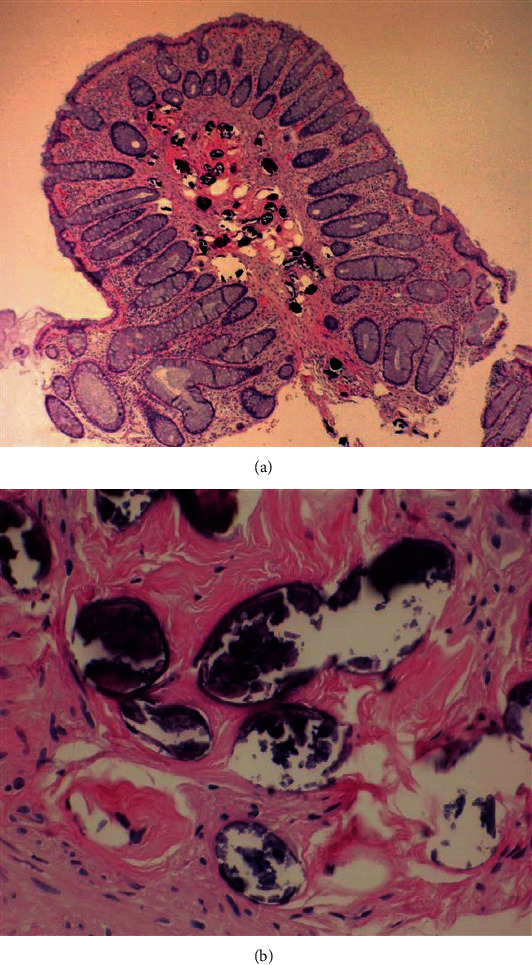
Biopsy specimen of a colonic polyp, which demonstrates several calcified *Schistosoma* eggs in the stroma of the polyp (a). Evidence of *Schistosoma* eggs within the colonic mucosa (b). (Image obtained with permission from Cerilli [59]).

There will be evidence of a thickened bowel wall with subsequent enteric cavity stricture and polyps within the left colon in advanced stages. A key finding is the presence of gray-yellow exudate similar to pseudomembranous colitis that may be present within the bowel wall. Regular histological sections can demonstrate Schistosome eggs, which can appear calcified or ruptured with infiltration of lymphocytes and plasma cells within the submucosa with a significantly higher macrophage distribution (Figure 7) [57]. There can also be evidence of yellow nodules in the biopsy specimen. Alternatively, crush preparations (crushing the biopsy specimen between two slides) can be obtained to visualize the Schistosome eggs better if they are not evident on regular histological specimens.

Intestinal Schistosomiasis can be treated with a 4- to 5-day course of praziquantel. Several studies in the literature have demonstrated that early active granulomas respond effectively to treatment. Still, therapy for chronic fibrotic or calcified granulomas will only minimize the inflammatory response and will not clear the pathogen [56].

#### 2.3.2. Anisakiasis

Anisakiasis is a fish-borne zoonosis and mostly occurs in countries where consumption of raw or inadequately cooked fish is high [60], namely, Japan, Northern Europe, Pacific Coast, and South America. Human Anisakiasis is caused by *A. simplex* and *A. pegreffii*. They live in the stomach of marine fish and mammals, and humans are accidentally contaminated. Once they infect humans, they are primarily found within the stomach, followed by the small intestine and colon. Pathogenesis of Human Anisakiasis occur in two ways [61]: an allergic reaction like angioedema, or gastroallergic Anisakiasis (primarily due to *A. simplex)* where patients may present with an acute abdomen, or it may infiltrate the tissue of the gut wall and lead to the formation of an eosinophilic granuloma and later perforation of the viscera. *A. simplex* is a frequent cause of urticaria and angioedema in Spain, and it is, in fact, a component of their standard food allergy and anaphylaxis testing. Gastric symptoms of Anisakiasis are vague, and acute infection can present as acute epigastric pain associated with nausea, vomiting, and bloody diarrhea. Anisakiasis's chronic form may lead to symptoms requiring surgical interventions, namely, abdominal peritonitis and intestinal obstruction.

**Figure 8:**
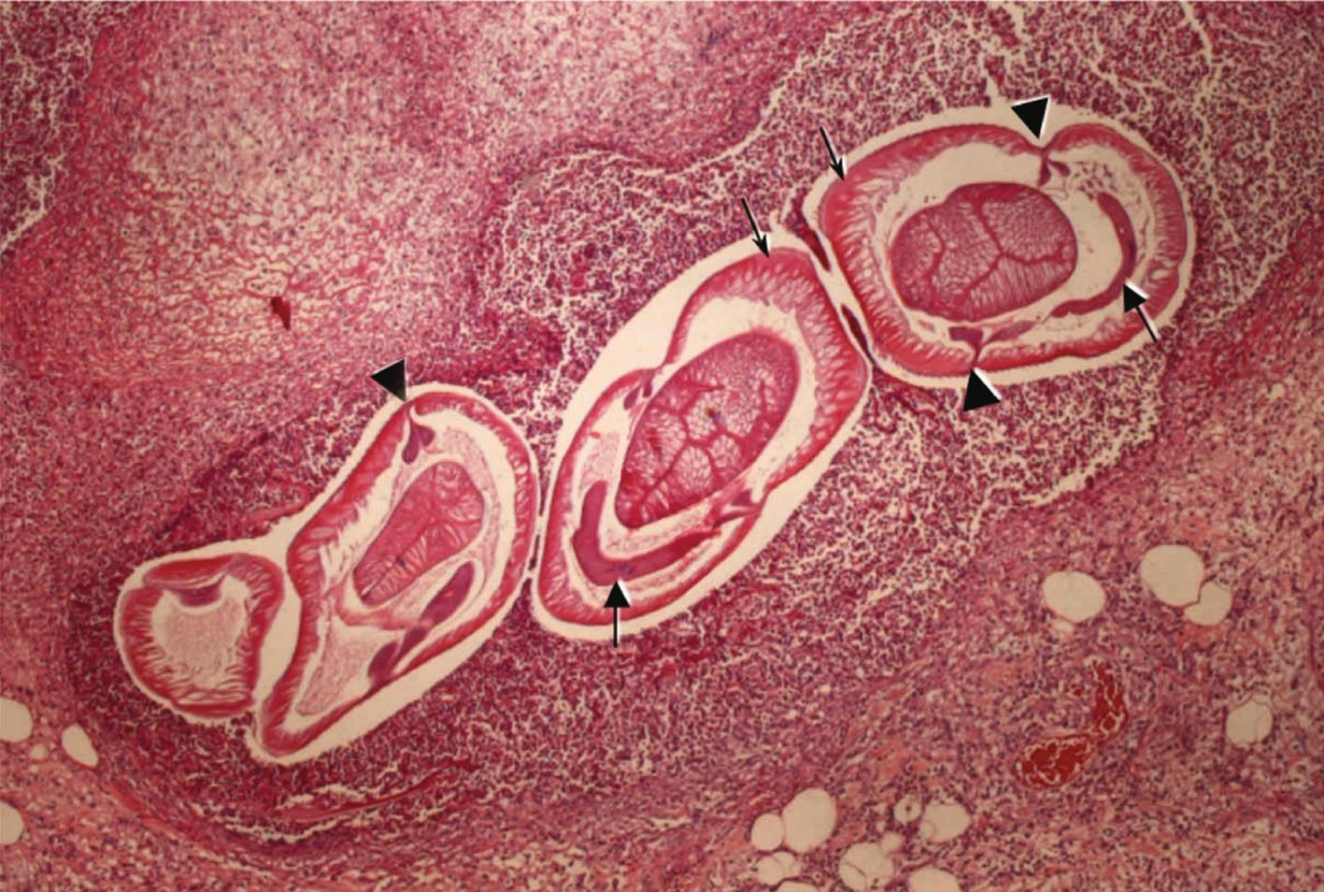
Transverse intestinal wall biopsy specimen, which demonstrates Anisakiasis larva within the submucosa. Thin arrows highlight polymyarial muscle cells, separated into ×4 quadrants by the chords demonstrating two wing-like lobes (arrowheads). Banana-shaped excretory cells are organized ventral to the intestine (thick arrows). (Image obtained with permission from Mattiucci et al. [62]).

Diagnosis can be made using patients' history and laboratory findings, including leukocytosis with eosinophilia. Consumption of raw fish within 72 hours is often the key to diagnosis. Real-time PCR is a more specific test for Anisakiasis than DNA sequencing [60]. Ultrasound may show large ascites with evidence of eosinophilia within the fluid and dilatation of the small intestine with localized edema of Kerckring folds [61]. Direct endoscopic visualization of the stomach and small intestine can reveal areas of hyperemic mucosa with superficial erosions and findings suggestive of granulomas [60]. Histological findings include ulcerated mucosa with granulomas with evidence of *Anisakis* within the muscular layer surrounded by histiocytes and eosinophils along with interstitial edema [61]. There are also reports that these findings can be evident transmurally to include the peri-intestinal fat tissue [62]. The larvae will appear as a thick multilayered cuticle with the organization of muscle fibers of polymyarial type in each quadrant of the worm with evidence of lateral chords with a unique butterfly-like appearance (Figure 8) [61].

### 2.4. Rare Causes of Infectious Granulomas of the Gastrointestinal Tract

#### 2.4.1. Bartonella henselae

This Gram-negative pleomorphic bacterium is the underlying cause of cat scratch disease in immunocompetent hosts and Bacillary angiomatosis in immunocompromised individuals, including those with AIDS and kidney or liver transplant recipients [63]. Cat scratch disease (CSD) is traditionally known to be transmitted via a male cat's bite or lick [28]. However, there are also reported cases of infection via dog bite and cat fleas, particularly *Ctenocephalides felis* [63, 64]. It presents with fever, erythematous papules, vesicular blisters, and regional lymphadenitis followed by necrotizing granulomatous lesions in the liver and spleen [63]. It is considered one of the key differentials in children less than ten years of age, presenting with fever of unknown origin and granulomatous hepatitis. Cases of CSD are often reported in North America during the late summer and fall seasons and predominantly in the pediatric population [63].

**Figure 9:**
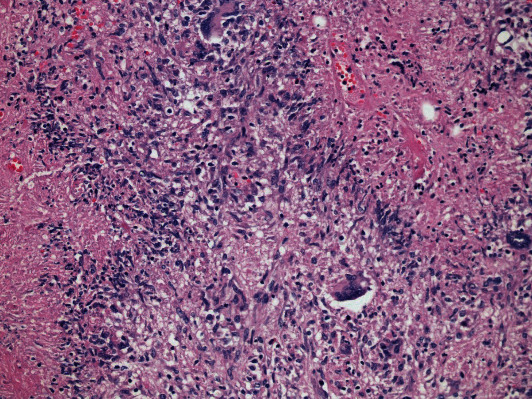
H&E biopsy specimen from a right partial hepatectomy shows necrotizing granulomas with giant cells and characteristic palisading histiocytes in an immunocompetent patient diagnosed with granulomatous hepatitis due to *Bartonella henselae* infection. (Image obtained with permission from VanderHeyden et al. [66].)

Clinical diagnosis includes the following criteria: history of contact with the target animal and presence of a scratch or lesions on the skin or eye (positive CSD skin test), exclusion of all other causes of lymphadenopathy, and the histologic finding of caseating granulomas in lymph node biopsies [65]. Target sites for CSD often include the small intestine, specifically the terminal ileum, and also the colon. However, for immunocompromised individuals, Bacillary angiomatosis in the GI tract can manifest as numerous nodules and ulcerations in the esophagus, stomach, duodenum, and colon. Hence, it is considered as a key differential for HIV patients presenting with multiple small ulcers throughout the GI tract. CT or high-resolution ultrasound can show evidence of lymphadenitis and hypodense lesions in the liver and spleen. One study reports that lymphadenitis can be severe enough to present as a mass protruding through the duodenal wall, therefore mimicking a malignant cause [64]. Biopsies demonstrate the presence of necrotizing granulomas with monoclonal B lymphocyte clusters adjacent to microabscesses. In Bacillary angiomatosis, the bacteria prefer to grow in between the collagen fibers or cluster around blood vessels (Figure 9) [28, 65]. Although CSD stains positive for the Warthin-Starry stain, it should be noted that it is relatively nonspecific and is often nondiagnostic [64, 65]. In such circumstances, PCR and ELISA tests can be used to demonstrate seropositivity for IgM antibodies, which remain elevated for up to 3 months after the onset of symptoms [64].

CSD is a self-limiting illness, and in some instances, the use of azithromycin or doxycycline is useful [63, 64]. Similarly, Bacillary angiomatosis is managed effectively via the use of erythromycin or tetracycline.

#### 2.4.2. Salmonellosis

*Salmonella typhi* is a Gram-negative bacterium that is responsible for the systemic infectious disease, typhoid fever. *S. typhi* only infects humans and is frequently diagnosed in travelers from endemic areas like India, Mexico, and the Philippines [67]. Primary sources of infection include poor sanitary conditions, contaminated water, meat, dairy, and egg products. The pathogen is a common cause of infectious granulomas in the liver, bone marrow, and spleen [67]. However, few substantial case reports describe granuloma formation within the GI tract itself that is caused by *S. typhi* [67, 68]. If the GI tract is involved, *S. typhi* usually targets the lymphoid tissue of the ileum, cecum, appendix, and right colon [67–69].

Symptoms of typhoid fever commonly appear 5–21 days after ingesting contaminated food or water. Symptoms often present in a progressive fashion over three weeks [67, 70]: fever rising over a few days along with relative bradycardia is common during week one; watery diarrhea, abdominal pain, and the classic “rose or salmon-colored” rash on the trunk and abdomen appear on week two; and on week three, watery diarrhea can become hemorrhagic and is often associated with intestinal perforation due to ileocecal lymphatic hyperplasia. Typhoid fever is not associated with high mortality if managed appropriately but can be fatal in the elderly, neonates, and immunocompromised individuals [70]. Hence, prevention is crucial, and travelers to endemic areas are advised to vaccinate before travel and are also educated to maintain food and water precautions [67].

**Figure 10:**
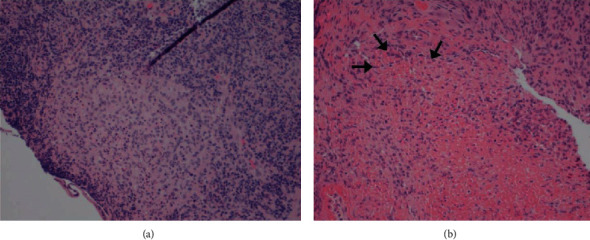
H&E stain of an ileal mucosal biopsy (a) and cecal mucosal biopsy (b) demonstrating histiocyte-rich granulomatous changes with central necrosis (arrows) in a patient diagnosed with granulomatous ileitis due to typhoid fever. (Images obtained with permission from Cheung et al. [67]).

The gold standard of diagnosis of *typhoid fever* is via cultures from the blood, stool, and urine [67]. However, blood culture sensitivity for typhoid fever is only 60-70%, and serology is nonspecific [68, 71]. Colonoscopic findings of the ileocolic region may include punched out mucosal ulcerations of various sizes that appear as either long, oval, or linear in shape, together with edematous, hyperemic mucosal patches. These ulcers can be deep enough to reach the muscularis layer and may show signs of bleeding or perforation [67, 69, 70]. A biopsy is vital for diagnosis. If granulomatous ileitis is suspected, then TB, Yersinia, and IBD need to be excluded since *S. typhi* is a common masquerader. Histological findings commonly include histiocytic-rich granulomas admixed with lymphocytes and plasma cells along with areas of central necrosis (Figure 10) [67, 69, 70]. Neutrophils are deficient in these specimens. The hallmark finding on biopsy is ulceration overlying the hyperplastic Peyer's patches that leads to ulcerated lymphoid follicles [69]. There can be evidence of marked architectural distortion and crypt abscesses. Crypt distortion is more prominent in IBD, and there is a paucity of chronic inflammatory cells in Salmonellosis [69]. TB can be ruled out using additional tests such as tuberculin skin test, PCR for *Mycobacterium* species, and special stains on biopsy specimens like Ziehl-Neelsen or AFB stains.

Treatment for typhoid fever is mostly supportive, along with the use of antibiotics such as fluoroquinolones. However, it must be emphasized that there is a growing concern for resistance to fluoroquinolones worldwide. Thus, in high-risk countries, the use of azithromycin or cephalosporins is the drug of choice [70].

#### 2.4.3. Syphilis

The anorectal region is the primary gastrointestinal site affected by syphilis (*Treponema pallidum* infection), although other sites can be involved, such as the stomach. Homosexual men are at high risk of disease. Anorectal syphilis is often underdiagnosed because of the variability of its clinical presentation. Patients are usually asymptomatic, but pain with defecation, constipation, bleeding, and discharge may be present in syphilitic proctitis. Luetic gastritis commonly manifests with upper GI bleeding, melena, and coffee-ground emesis, along with nonspecific symptoms of nausea, fever, malaise, anorexia, early satiety, and epigastric pain [72].

Endoscopic findings of syphilitic anorectal disease are broad, ranging from proctitis and ulcers to pseudotumors [73]. Ulcers can be irregular and numerous, and two ulcers can be found, opposing one another, or can be eccentrically located [74]. After an incubation period of three weeks, a papule may develop at the inoculation site instead of these atypical ulcers. The papule can later evolve into an ulcer with raised margins that are indurated with a nonexudative base, resembling the typical chancre of primary syphilis. Histological specimens are characterized by dense mononuclear cell infiltrates with prominent plasma cells, accompanied by cryptitis, crypt abscesses, glandular destruction, and reactive epithelial changes. It is occasionally associated with granulomas in isolated case reports.

**Figure 11:**
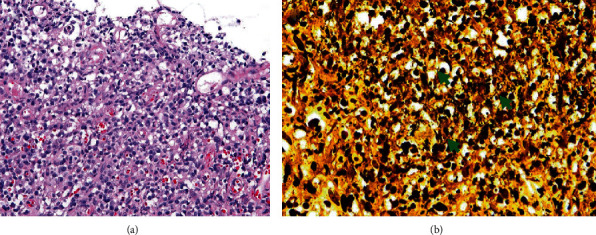
Histopathologic findings demonstrate a rectal ulcer with inflammation with a predominance of plasma cells (a). Warthin-Starry stain demonstrates numerous spirochetes with spiral rods (arrows in (b)). (Images obtained with permission from You et al. [76]).

Additionally, proliferative endarterteriologitis (prominent proliferative capillary endothelial cells) may be observed [75]. Syphilitic proctitis can be nonspecific, demonstrating features of acute self-limited colitis or focal colitis with or without an increase in plasma cells (Figure 11) [72]. Histological specimens of syphilitic gastritis often demonstrate dense plasmacytic infiltrates. Glands may be spared of inflammation, and fibrosis can be prominent as disease progresses. The endoscopist should perform multiple biopsies as histological evaluation can rarely be nonspecific.

#### 2.4.4. Basidiobolomycosis

Basidiobolomycosis is a rare chronic fungal infection caused by *Basidiobolus ranarum* and is common in temperate climates, namely, Brazil, Iran, India, Saudi Arabia, and Southwest United States of America [77]. It mostly affects immunocompetent adults via the ingestion of food contaminated with infected soil or animal feces (bats, reptiles, or fish). GI involvement includes the small intestine, colon, and rectum in 80% of cases, and 20% of cases involve the liver. Gastrointestinal Basidiobolomycosis may present with fever, abdominal pain, constipation or bloody diarrhea, or a mass-like lesion within the colon resembling colonic malignancies [78, 79]. The most frequent complication includes intestinal obstruction.

**Figure 12:**
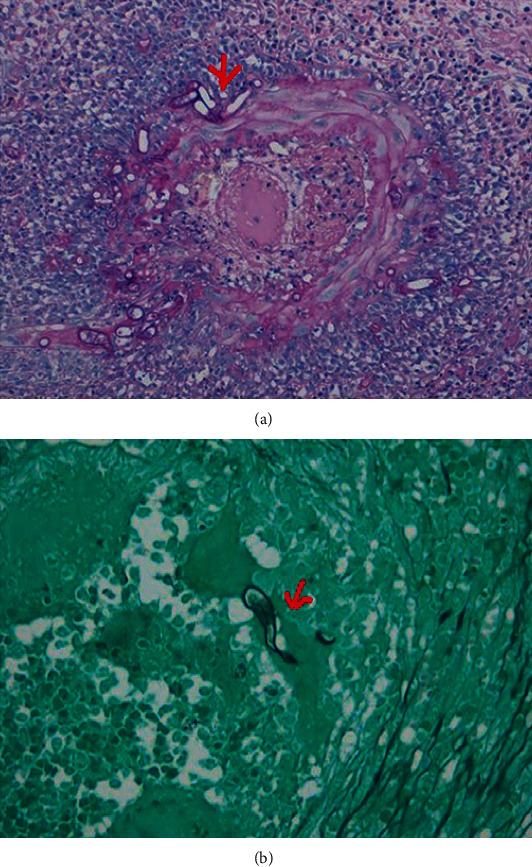
A small intestine biopsy specimen shows the Splendore-Hoeppli phenomenon in a patient diagnosed with colonic Basidiobolomycosis ((a) red arrow). Grocott stain of the small intestine shows Basidiobolus hyphae (red arrow), which appears thin-walled, septated hyphae surrounded by eosinophilic material (b). (Images obtained with permission from Kurteva et al. [81]).

Laboratory findings may reveal eosinophilia. Colonoscopy shows superficial ulcers within the cecum and ileocecal valve. Deep biopsies are required as they reside within the submucosa and show necrotizing granuloma with eosinophilic infiltration and Splendore-Hoeppli phenomenon characterized by the presence of amorphous, eosinophilic hyaline material that surrounds the organism (Figure 12) [78]. Fungal cultures are the gold standard of diagnosis and will show yellow colonies with characteristic waxy growth. Itraconazole is the most frequently used agent, followed by amphotericin, ketoconazole, and voriconazole [80].

#### 2.4.5. Leishmaniasis

Leishmaniasis is a vector-borne protozoan infection spread by the bite of a female sandfly, genus *Phlebotomus*, or genus *Lutzomyia* species [82]. This disease is endemic to the tropics and subtropics, particularly Southeast Asia and East Africa. More specifically, *L. donovani* is predominant in Asia and Africa, while *L. infantum* is frequent in Europe [32].

Initial manifestations can either be cutaneous, mucocutaneous, or visceral, which alone carries a mortality rate of 75-92% [82]. *L. donovani* and *L. infantum/chagasi* are mostly responsible for Visceral Leishmaniasis (VL), which is more prolifically known as Kala-Azar [32]. They affect the lungs, pleura, larynx, esophagus, stomach, small intestine, bone marrow, and skin [82]. VL manifesting in the GI tract alone is only apparent in immunosuppressed individuals, mostly HIV patients, and is rare in healthy individuals [82].

**Figure 13:**
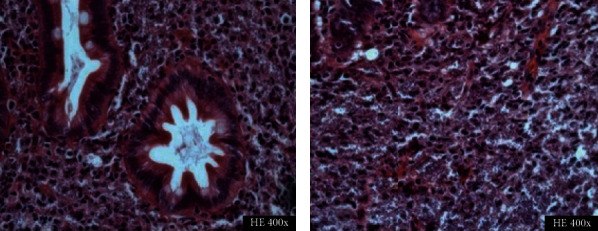
Biopsy specimen of the duodenum of a patient diagnosed with Visceral Leishmaniasis demonstrates extensive infiltration with *Leishmania* bodies. (Images obtained with permission from Cota et al. [84]).

Symptoms are often nonspecific ranging from fever, weight loss, hepatosplenomegaly to pancytopenia secondary to bone marrow involvement, to hypergammaglobulinemia [32]. However, it is imperative to have a high index of suspicion in travelers from these endemic regions presenting with refractory diarrhea. Endoscopy shows atrophy of the duodenal mucosa, and biopsy will demonstrate evidence of infiltration of Leishman-Donovan bodies with neutrophils and histiocytes (Figure 13) [83]. It has been reported that these parasites are plentiful in the spleen and liver especially in those with hepatosplenomegaly [82]. The most useful diagnostic test is a bone marrow smear, but in some cases, a splenic aspirate will suffice since it has a sensitivity of 98% [32, 82]. Noninvasive testing can include enumerating antibodies to Leishmania via direct agglutination (DAT) and serological antibody to rK39 (polypeptide of Leishmania) tests, and both have sensitivities of 95% and 94%, respectively [82].

Treatment involves the use of pentavalent antimonials, sodium stibogluconate, amphotericin B, miltefosine, paromomycin, and pentamidine. Miltefosine is predominantly used in developing countries for its cost-effectiveness, and amphotericin B is useful in resistant cases. Novel treatment involving liposomal amphotericin B is well tolerated with fewer side effects considering its high therapeutic index.

## 3. Conclusion

This review article provides a detailed overview of the commonly encountered infectious causes of granulomatous gastrointestinal tract diseases, amidst an extensive list of etiologies. The report also focuses on the most up-to-date information and offers diagnostic clues in the realms of clinical history, imaging (radiographic and endoscopic), and biopsy as well as the appropriate treatment for each pathology. We hope that this near-exhaustive list will prove useful for gastroenterologists and pathologists.

## Figures and Tables

**Table 1:** Comparison of imaging and microscopic findings together with regions of the gastrointestinal tract affected by bacterial causes of granulomatous disease.

Bacterial granulomatous etiologies	Location in the gastrointestinal tract	Imaging findings	Histological findings
Mycobacterium tuberculosis	Ileocecum → ascending colon → jejunum → appendix → duodenum → stomach → esophagus → sigmoid colon → rectum	Endoscopy (i) 3 main types: ulcerative, hypertrophic, and ulcerohypertrophic (ii) pseudopolyps, nodules, transverse ulcers, and a deformed ileocecal valve may be present CT/MRI (i) Concentric wall thickening, agape ileocecal valve, and strictures with prestenotic dilation	(i) Table 4 provides a detailed comparison of the histopathological features of *M. tuberculosis* and Crohn's disease Briefly (i) Multiple, large granulomas per high power field (ii) Caseating granulomas, with confluence, prominent lymphoid cuff, and architectural distortion (iii) Epithelioid histiocyte ulcers, submucosal granulomas, lymphocyte cuffing, and excessive submucosal inflammation (iv) AFB+

Bartonella henselae	Cat scratch disease (i) Terminal ileum and colon Bacillary angiomatosis (i) Esophagus, stomach, duodenum, and colon	Endoscopy (i) Multiple small ulcers throughout the GI tract in bacillary angiomatosis CT abdomen (i) Lymphadenitis (may present as protruding mass) and hypodense lesions in the liver and spleen	(i) Caseating granulomas with monoclonal B-cell clusters and microabscesses (ii) In Bacillary angiomatosis, this finding together with the growth of bacteria in between collagen fibers or cluster around blood vessels (iii) Warthin-Starry stain+

Yersinia bacterium	Terminal ileum, cecum, appendix, and mesenteric lymph nodes	Endoscopy (i) Acute inflammation with purulent and necrotic lymphadenopathy in distal ileum and cecum CT abdomen (i) Evidence of pseudotumor, colitis, ulceration, and aphthoid ulcers in the region of the terminal ileum with or without mesenteric lymphadenopathy and appendiceal inflammation	(i) Numerous, large, suppurative epithelioid granulomas without evidence of monoclonal B cells with microabscesses (ii) *Y. pseudotuberculosis* in later stages may present with neutrophilic invasion and scattered microabscesses with pus surrounded by a suppurative granuloma (iii) Histology similar to Crohn's disease may be present: transmural inflammation and cryptitis

Lymphogranuloma venereum	Rectum and distal colon	Endoscopy (i) Like IBD, it will show chronic inflammatory changes with strictures and ulcerative lesions in the mucosa covered with pus, blood, and granulation tissue CT abdomen (i) Similar to IBD and will demonstrate fibrotic tissue, strictures, and fistulas in the colorectal region	(i) Acute inflammation stage may show cryptitis and crypt abscesses with minimal crypt distortion (ii) The chronic inflammatory phase will show nonsuppurative epithelioid granulomatous inflammation with focal necrosis, numerous crypt abscesses, and without evidence of monoclonal B cells

Syphilis	Most commonly targets anus and rectum; stomach occasionally involved	Endoscopy (i) Findings can range from proctitis to ulcers (numerous and irregular) and pseudotumors (ii) In the early stages, a papule can be present within the mucosa of the anus/rectum and can later evolve into an ulcer resembling a typical chancre of primary syphilis	(i) Dense mononuclear cells with prominent plasma cells along with evidence of cryptitis, crypt abscess, and glandular destruction (ii) Proliferative endarterteriologitis can be present (iii) Syphilis is occasionally associated with caseating granulomas
Salmonellosis	Ileum, cecum, appendix, and right colon	Endoscopy (i) Hyperemic mucosal patches with punched out mucosal ulcerations of various sizes and shapes (long, oval, or linear) (ii) Deep ulcers can reach the muscularis layer with risk of bleeding and perforation	(i) Hallmark finding—ulceration overlying hyperplastic Peyer's patches leading to ulcerated lymphoid follicles (ii) Histiocytic-rich granulomas (rare) admixed with lymphocytes and plasma cells in addition to areas of central necrosis. These specimens are deficient of neutrophils (iii) Marked architectural distortion and crypt abscesses (iv) IBD, TB, and Yersiniosis must be ruled out first as Salmonellosis is a common masquerader

**Table 2:** Comparison of imaging and microscopic findings together with regions of the gastrointestinal tract affected by fungal causes of granulomatous disease.

Fungal granulomatous etiologies	Location in the gastrointestinal tract	Imaging findings	Histological findings
Histoplasmosis	Ileocecal junction → colon, small intestine, and upper GI tract	Endoscopy (i) Friable polypoid mass with focal erosion ranging from terminal ileum to rectum CT abdomen (i) Polypoid and apple core lesions with regional lymphadenopathy (ii) Colonic skip lesions	(i) Clusters of macrophages localized in the lamina propria forming caseating granuloma-like lesions, containing engulfed intracellular yeast with narrow budding which is PAS+ and GMS+ (ii) It can also present as noncaseating granulomas but appear fewer in number (<2 granulomas per slide) with ragged borders. This is in contrast to Sarcoidosis, which demonstrates numerous granulomas (>10 per slide) with clearly defined, sharp borders

Cryptococcosis	Esophagus, stomach duodenum (high preference)	Endoscopy (i) A nonspecific patchy, friable, and erosive lesion with swollen villi in the duodenum	(i) Caseating granulomas with a moderate histiocytic response and minimal recruitment of lymphocytic and neutrophilic components with evidence of intracellular narrow budding yeast which stains positive for PAS, GMS, and Alcian blue

Coccidioidomycosis	Peritoneum	CT abdomen (i) Omental thickening with areas of loculated ascites Diagnostic laparoscopy (i) Multiple white plaques throughout peritoneum and omentum	(i) Caseating suppurative granulomas with giant cells with multinucleated thick-walled spherules containing endospores and stain positive for PAS and GMS (ii) *Coccidioides* spp. can be distinguished from *H. capsulatum* and *Cryptococcus* spp. on the basis of variation in the size of the endospore and growing spherule as well as lack of narrow-based budding

Basidiobolomycosis	Intestines and rectum (80%) and liver (20%)	Endoscopy (i) Superficial ulcers within the cecum and ileocecal valve	(i) Deep biopsy required as the pathogen buries itself within the submucosa (ii) Necrotizing granuloma with eosinophilic infiltration and Splendore-Hoeppli phenomenon (presence of amorphous, eosinophilic, and hyaline material surrounding the organism)

**Table 3:** Comparison of imaging and microscopic findings together with regions of the gastrointestinal tract affected by parasitic causes of granulomatous disease.

Parasitic granulomatous etiologies	Location in the gastrointestinal tract	Imaging findings	Histological findings
Schistosomiasis	Small (*S. japonicum*) and large intestines (*S. mansoni*) and liver	Endoscopy (i) Early-stage: edematous mucosa with superficial ulcers and petechial hemorrhage most commonly seen in the right colon (ii) Advanced stage: thickened bowel wall with strictures and polyps mostly within the left colon. Presence of gray-yellow exudates on the bowel wall	(i) Regular biopsies will demonstrate Schistosome eggs. If not clearly evident and there is a high index of suspicion, then crush biopsies can be diagnostic (ii) Early-stage: Schistosoma ova within the lamina propria with infiltration of the submucosa with eosinophils and neutrophils (iii) Advance stage: calcified or ruptured ova within the granuloma with infiltration of macrophages, lymphocytes, and plasma cells within the submucosa

Anisakiasis	Stomach → small and large intestines	Endoscopy (i) Erythematous mucosa with superficial erosions and granulomas Ultrasound (i) Large ascites with eosinophilia within the ascitic fluid (ii) Dilation of the small intestine with localized edema of Kerckring fold	(i) Ulcerated mucosa with granulomas along with evidence of *Anisakis* within the muscular layer surrounded by histiocytes and eosinophils (ii) Larvae will appear as thick multilayered cuticles with muscle fibers of polymyarial type in each quadrant of the worm and evidence of lateral chords with a characteristic butterfly-like appearance

Leishmaniasis	Esophagus, stomach, and small intestine	Endoscopy (i) Atrophy of the duodenal mucosa	(i) A granulomatous lesion with neutrophils and histiocytes with infiltration of Leishman-Donovan bodies

**Table 4:** Comparison of histological features between *Mycobacterium tuberculosis*, *Yersinia*, and Crohn's disease [2, 75].

Histopathological features	Tuberculosis	Yersinia spp.	Crohn's disease
Number of granulomas	Numerous	Numerous	Few
Size of granulomas	Large (>200 *μ*m)	Large	Small (<200 *μ*m)
Other granuloma features:			
(i) Caseating	Common	Few	Absent
(ii) Confluence	Common	Common	Absent
(iii) Prominent lymphoid cuff	Common	Common	Uncommon
(iv) Lymphoid hyperplasia	Common	Very common	Uncommon
(v) Architectural distortion	Common	Common	Common
(vi) Ulcers (deep and aphthous)	Common	Common	Common
(vii) Changes of chronicity unassociated with sites of granulomatous inflammation	Absent	Absent	Common
Multiple sites of involvement	Common	Rare	Common
Cobble-stoning of mucosa	Uncommon	Uncommon	Common
Fistulas	Uncommon	Rare	Common
Anal or perianal disease	Rare	Absent	Common
